# Music therapy and pharmacotherapy as a combination treatment: a case of periodic depression in comorbidity with subthreshold autism

**DOI:** 10.1192/j.eurpsy.2025.1340

**Published:** 2025-08-26

**Authors:** H. N. Lund, A. Drago

**Affiliations:** 1 Unit for Depression, Aalborg University Hospital, Psychiatry, Aalborg, Denmark

## Abstract

**Introduction:**

This case study investigates the combined use of pharmacotherapy and music therapy in treating a 44-year-old male patient with recurrent depression and subthreshold autism traits. These traits contributed to emotional rigidity and reduced treatment outcomes, requiring an interdisciplinary approach to enhance treatment effects.

**Objectives:**

To explore the efficacy of pharmacotherapy and music therapy in treating recurrent depression complicated by subthreshold autism traits, focusing on emotional regulation and coping strategies in a neuroatypical patient.

**Methods:**

The patient was treated in outpatient psychiatry with citalopram (10 mg/day) and nortriptyline (100 mg/day) while attending 18 months of individual music therapy at Aalborg University Hospital. The music therapy involved listening and improvisation aiming at addressing neuroatypical emotional rigidity and sensitivity.

**Results:**

While pharmacotherapy alleviated depressive symptoms, music therapy enabled expression and management of difficult emotions improving emotional flexibility and enhancing coping strategies. The patient was not diagnosed with Asperger’s syndrome, but exhibited traits of autism that influenced the treatment response.

**Conclusions:**

The combination of pharmacotherapy and music therapy proved beneficial for the patient, offering a non-verbal approach to emotion regulation. This case highlights the value of interdisciplinary approaches for treating depression in patients with subthreshold autism, especially in complex, treatment-resistant cases.

**Disclosure of Interest:**

None Declared

